# An unusual single paraglottic space neurofibroma: A case report and literature review

**DOI:** 10.1016/j.ijscr.2022.107286

**Published:** 2022-06-07

**Authors:** Sofia Anastasiadou, Gurpreet Sandhu

**Affiliations:** Imperial College NHS Trust, ENT Department, Charing Cross Hospital, London, United Kingdom of Great Britain and Northern Ireland

**Keywords:** Paraglottic schwannoma, Paraglottic neurofibroma, Nerve sheath tumours

## Abstract

**Background:**

Neurofibromas are benign peripheral nerve sheath tumours, formed by a wide variety of different kinds of cells and matrixes. Airway neurofibromas are extremely rare and minimal cases have been described in the literature. Endoscopic surgical removal is considered challenging due to the infiltrating character of the lesion.

**Case presentation:**

We describe the clinical case of a gentleman, with a left paraglottic space neurofibroma, who presented with dyspnoea and voice changes. He had his neurofibroma removed endoscopically under general anaesthesia.

**Methods:**

The case was analysed and discussed on this gentleman's presentation. However, the diagnosis was set only after the histology report was released. A literature review was performed to explore presentation and management.

**Conclusion:**

Neurofibromas have extremely low prevalence in the airway. The size of the benign tumour in our case was impressive and the recovery of the gentleman post-operatively was rapid.

## Highlights

• Neurofibromas are benign peripheral nerve sheath tumours, formed by a different kinds of cells and matrixes.
• In ENT, neurofibromas can be found on the tongue, buccal mucosa, palate, lips and gingiva as well as on the oropharynx.
• Clinical symptoms and findings depend massively on the location of the neurofibromas, their shape and size
• Multidisciplinary team recovery with speech and language therapists, ENT doctors and voice specialists is critical recovery of the patient.

## 1 Introduction

Neurofibromas are benign peripheral nerve sheath tumours, formed by a wide variety of different kinds of cells such as fibroblasts, Schwann cells, mast cells and perineural cells. Apart from cells, they also consist of a mixture of collagen and extracellular matrix. Neurofibromas can occur on any tissue and they can be variable in shape and size. In most cases, neurofibromas present in the context of neurofibromatosis type 1, an autosomal dominant disorder. However, they can be found as solitary, thus their incidence is unclear. While head and neck skin neurofibromas are very common reaching 51 % of patients with neurofibromas, airway neurofibromas are extremely rare and minimal cases have been described in the literature.

## 2 Case presentation

We describe the presentation of a 36 year old gentleman, who presented in outpatients' clinic with increasing difficulty in breathing and audible stridor. He has been having the symptoms for 6 months but aggravating and recently they have deteriorated. He was having difficulty mainly in inspiration and the stridor was as well inspiratory. He was also experiencing severe voice changes, suffering from significant voice fatigue and considerable fluctuance in voice quality. The patient was getting concerned regarding his condition as both voice and breathing were being affected. He had no other symptoms, he was able to swallow, eat and drink normally and had no signs of infection or inflammation. Symptoms were constant and severe, not aggravating or improving and were preventing him from completing his normal life activities. He was also about to have his first child, a fact that was distressing him even more to get quick and effective cure of his condition.

### 2.1 Investigations

**Fig. 1:**
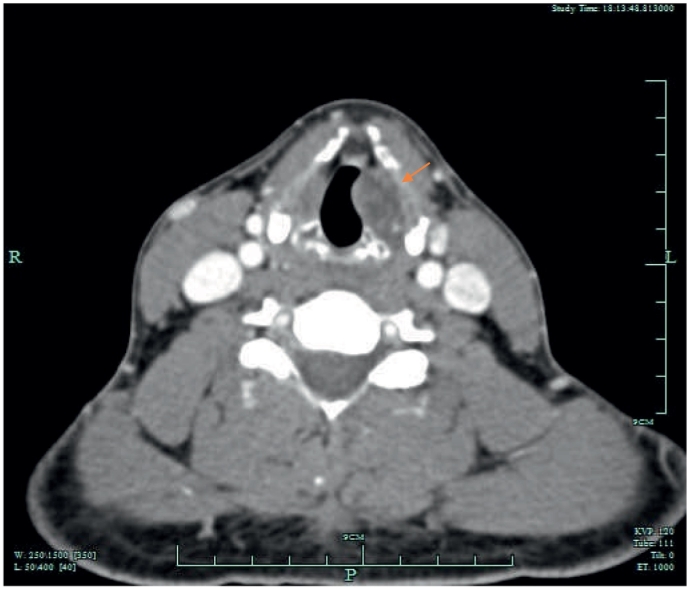
CT scan of the lesion.

**Fig. 2:**
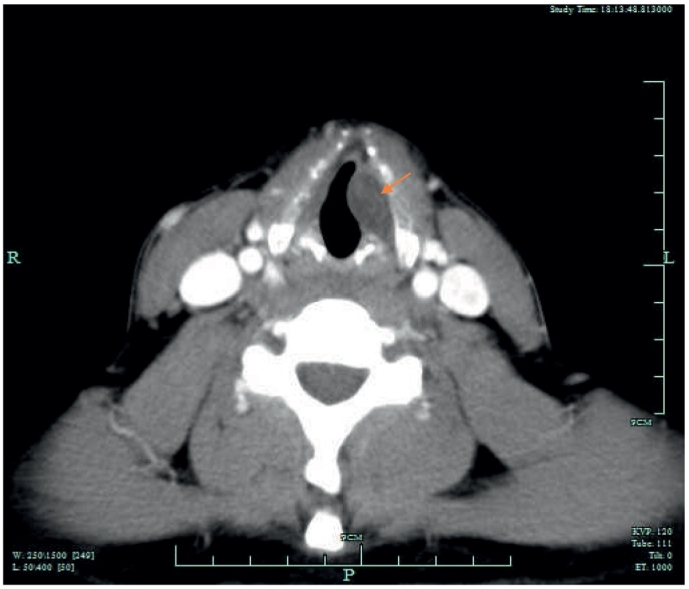
CT scan of the lesion.

**Fig. 3:**
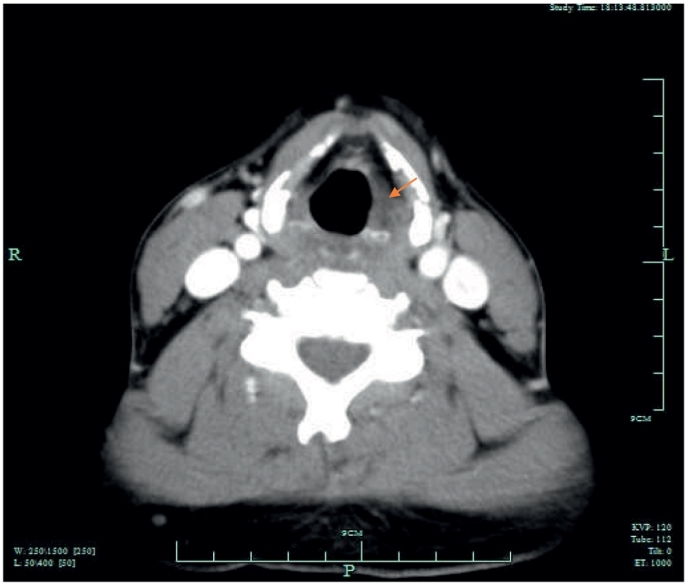
CT scan of the lesion.

**Fig. 4:**
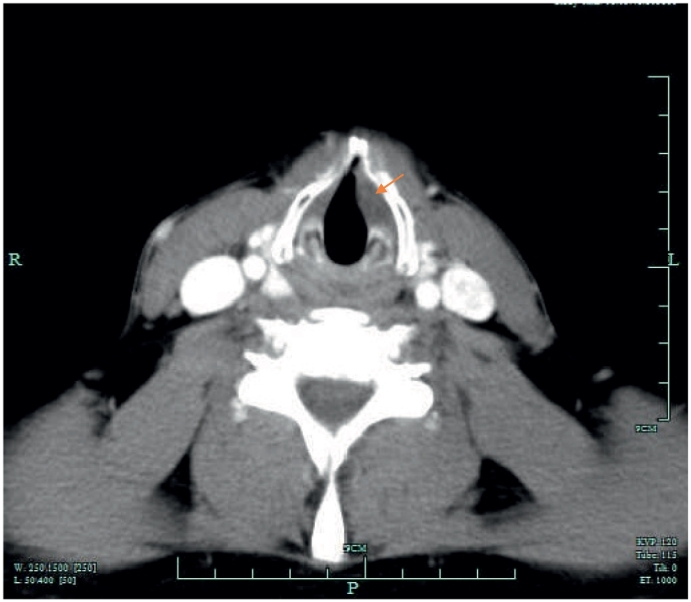
CT scan of the lesion.

**Fig. 5:**
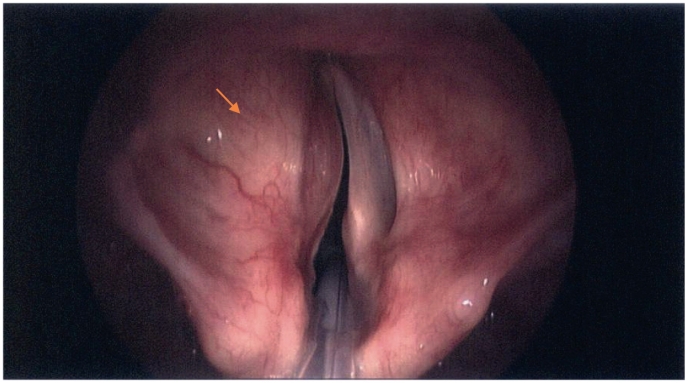
Pre excision.

**Fig. 6:**
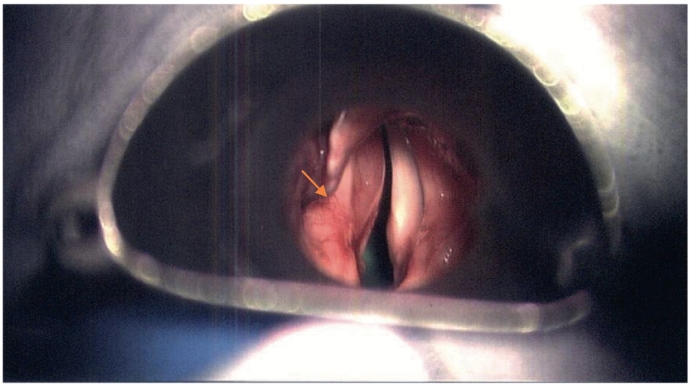
Endoscopic view.

On direct laryngoscopy, a 22 mm left sided vallecular lesion was identified, causing partial airway obstruction. The lesion was extending to the subepithelial area of the left vocal cord and the underlying muscle was thinned (Fig. 5, Fig. 6). On examination, there was no mass on neck palpation and no other clinical findings were present. Past medical history of the patient included only hypertension and reflux. The mass was subsequently assessed with a Computed Tomography of the neck and a neural sheath tumour was suspected radiologically (Fig. 1, Fig. 2, Fig. 3, Fig. 4). Before excising the lesion, the patient was made aware that due to the mass's expansion, there would be a wide excision that could cause to him more severe voice problems and that a second operation might be needed to restore their voice. This would have been a medialisation laryngoplasty in case his vocal cord function was disrupted permanently. It was also explained that malignancy could not be ruled out.

**Fig. 7:**
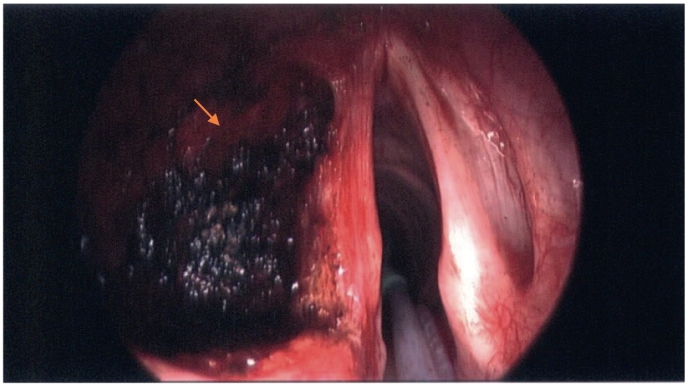
Post laser excision.

**Fig. 8:**
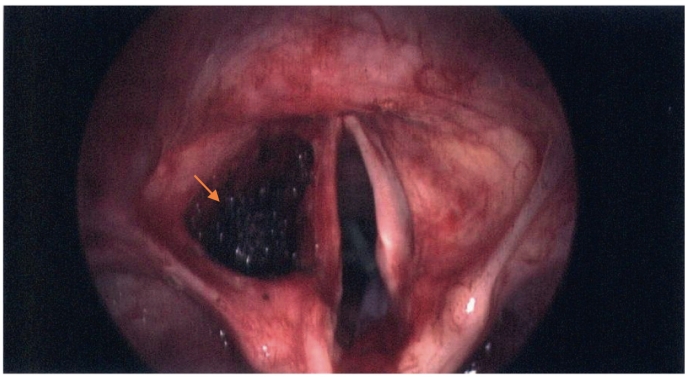
Post laser excision.

### 2.2 Treatment

The patient had anaesthetic pre-assessment and found to be eligible for a general anaesthetic. He was given a general anaesthetic and direct laryngoscopy was performed to re-assess the lesion. Microlaryngoscopy was essential as laser was the treatment mode of choice. The mass was excised with the laser endoscopically using a wide approach through the supraglottic larynx into the paraglottic space (Fig. 7, Fig. 8). The specimen was sent for analysis, bleeding was minimal, and patient had an uneventful recovery. Eventually, the histology report showed a schwannoma.

### 2.3 Outcome and follow up

The patient was assessed and followed up after the operation until today. Although he recovered rapidly after the operation, his voice change and fatigue were persistent even six months after the operation. The voice quality was preventing him from day to day activities and took him more than three months to return back to work, even with minimal voice use. Although the actual mass had not recurred, persistent inflammation of the left vocal cord was preventing him from having a normal quality voice. He was having bowing of the left vocal cord and a significant phonatory gap post-operatively. A medialisation laryngoplasty was considered to improve his voice outcome, which was discussed with him. However, speech therapy was started, and his voice strength increased significantly after that. His last visit in clinic demonstrated a normal vibration and approximation of the vocal cords on stroboscopy. Therefore, excision was complete and successful and post-operative outcome was overall satisfactory.

## 3 Discussion

Neurofibromas are nerve sheath tumours that can be encountered either isolated either in groups normally found in systematic diseases. There is no predominantly affected population and the presence of neurofibromas is not affected by age, sex or other predisposing factors. There are two different sub-groups of neurofibromas: dermatomal and plexiform with the latter ones to present prominently to the head and neck region [4]. Clinical symptoms and findings depend massively on the location of the neurofibromas, their shape and size and also the involvement of surrounding tissues. Schwannomas are usually found as an extension to the nerve while neurofibromas grow incorporating the surrounding structures. Microscopically, they present as non-encapsulated spindle cell lesions, maintaining intact squamous cell epithelium and keeping their nuclei atypia low without clear signs of malignancy.

Ear nose and throat surgery rarely involves isolated neurofibromas [5]. There are many cases of head and neck neurofibromas in the context of a systematic disease such as neurofibromatosis [3]. These lesions can be found on the tongue, buccal mucosa, palate, lips and gingiva as well as on the oropharynx. However, isolated paraglottic neurofibromas are very rare and only one more vallecular neurofibroma was found reported in literature. In addition to that, one more nerve sheath lesion has been reported in literature located on the patient's soft palate. A cheek and an oropharyngeal neurofibroma have been also reported and were both excised successfully [6], [7]. Imaging involves CT scans, MRI scans and direct laryngoscopy or microscopic laryngoscopy.

There are different approaches in treating paraglottic masses such as excision, laser excision, coblation and photodynamic therapy as a primary intervention [8]. In our case, deep laser was used that excised the lesion completely but also led to slow voice recovery that was eventually restored successfully with speech therapy. Other therapies include coblation of the lesion [4] and also intralesional excision which is highly associated with recurrence [9], [10]. Nevertheless, intralesional excision encounters the risk of sacrificing surrounding structures that leads to poor aesthetic and voice outcome.

Even though recurrence of neurofibromas after complete excision is rare, continuous follow up and regular outpatient appointments are essential. Multidisciplinary team recovery with speech and language therapists, ENT doctors and voice specialists is also critical in order to ensure safe and effective recovery of the patient.

This case report was written according to SCARE guidelines [11].

## Consent

Written informed consent was obtained from the patient for publication of this case report and accompanying images. A copy of the written consent is available for review by the Editor-in-Chief of this journal on request.

## Ethical approval

No ethical approval was required.

## Funding

No funding was required for this research paper.

## Guarantor

Ms Sofia Anastasiadou.

## Research registration number

It is also registered in the researchregistry.com/with the unique number: researchregistry7975.

## CRediT authorship contribution statement

Both authors took care of each section of the paper and worked together.

## Declaration of competing interest

Authors declare no conflict of interest.
